# Supplementary material for: KDM2B controls HIF levels and activity through its JmjC and CxxC domains
Source: Biochem J. 2026 Sep 17;483(10):1979–97. doi: 10.1042/BCJ20260212 (PMC13591259; doi:10.1042/BCJ20260212)
Supplement: Supplementary file 1 [file BCJ-2026-0212_supp.pdf]

Supplementary

Batie et al., KDM2B controls HIF levels and activity through its JmjC and CxxC domains

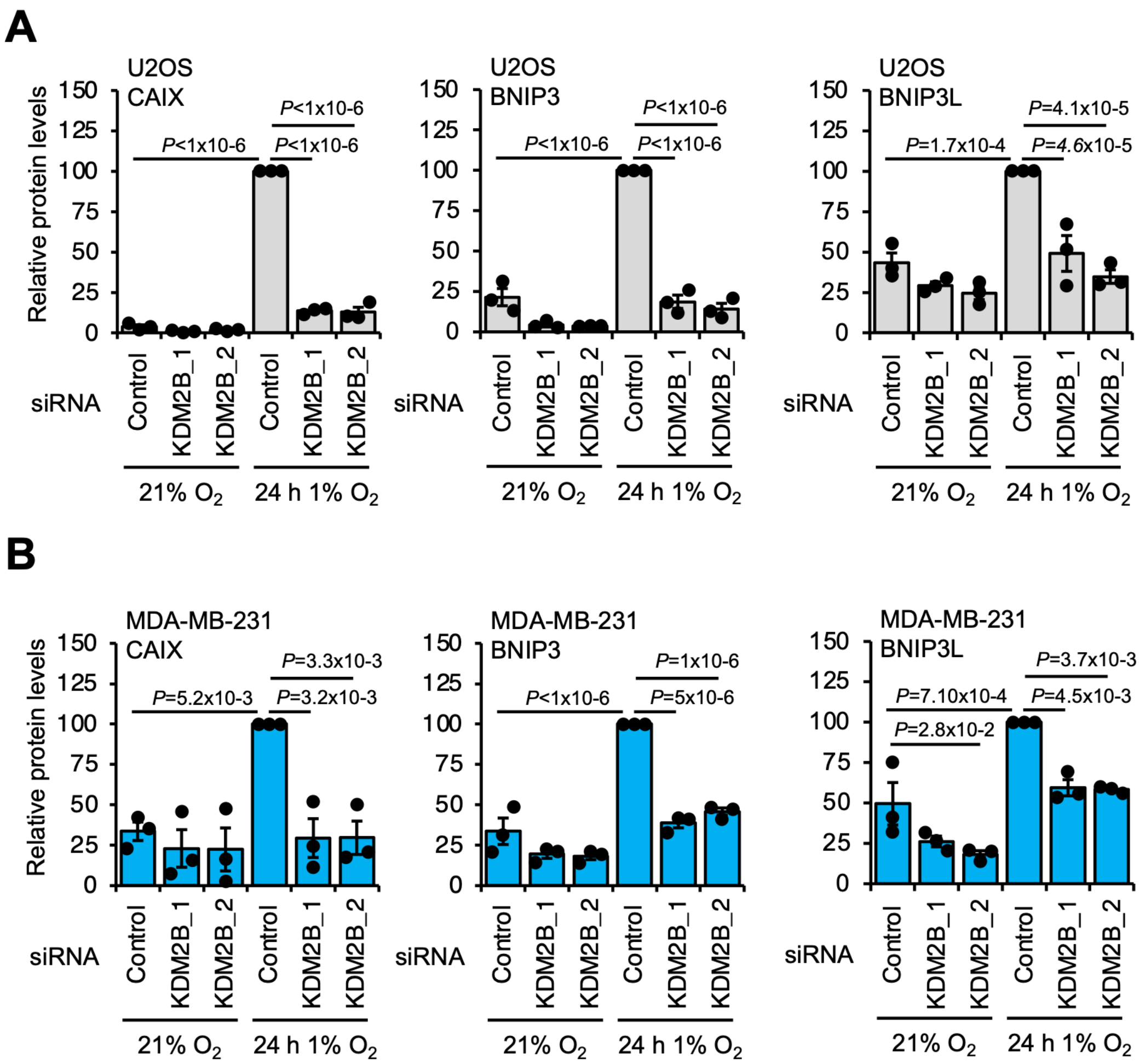

Supplementary Figure S1. Regulation of HIF activity by KDM2B in U2OS and MDA-MB-231 cells, Western blot quantification. Western blot quantification analysis for the indicated proteins in **A)** U2OS and **B)** MDA-MB-231 cells cultured at 21% oxygen, transfected with control, KDM2B_1, or KDM2B_2 siRNA for 48 hours, and exposed or not to 1% oxygen for 24 hours. β-actin was used as a normalising protein. Data represent mean (n=3 biological replicates) ± SEM. *P* values for group comparisons were calculated by one-way ANOVA with Tukey's test.

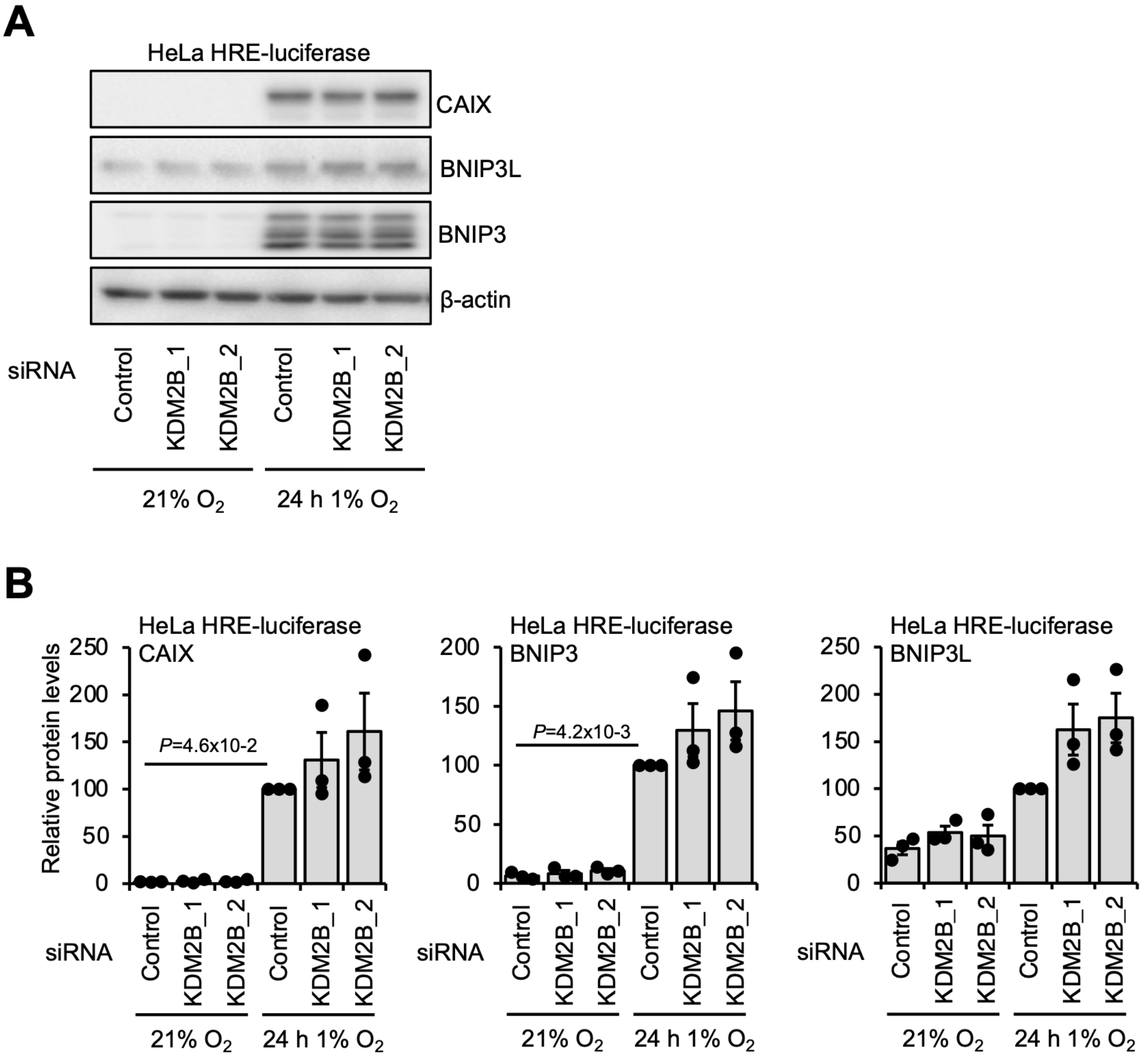

Supplementary Figure S2. Regulation of HIF activity by KDM2B in HeLa HRE-luciferase cells. **A)** Western blot and **B)** Western blot quantification analysis for the indicated proteins in HeLa HRE-luciferase cells cultured at 21% oxygen, transfected with control, KDM2B_1, or KDM2B_2 siRNA for 48 hours, and exposed or not to 1% oxygen for 24 hours. β-actin was used as a normalising protein. A representative Western blot of three biological replicates is shown. Data represent mean (n=3 biological replicates) ± SEM. *P* values for group comparisons were calculated by one-way ANOVA with Tukey's test.

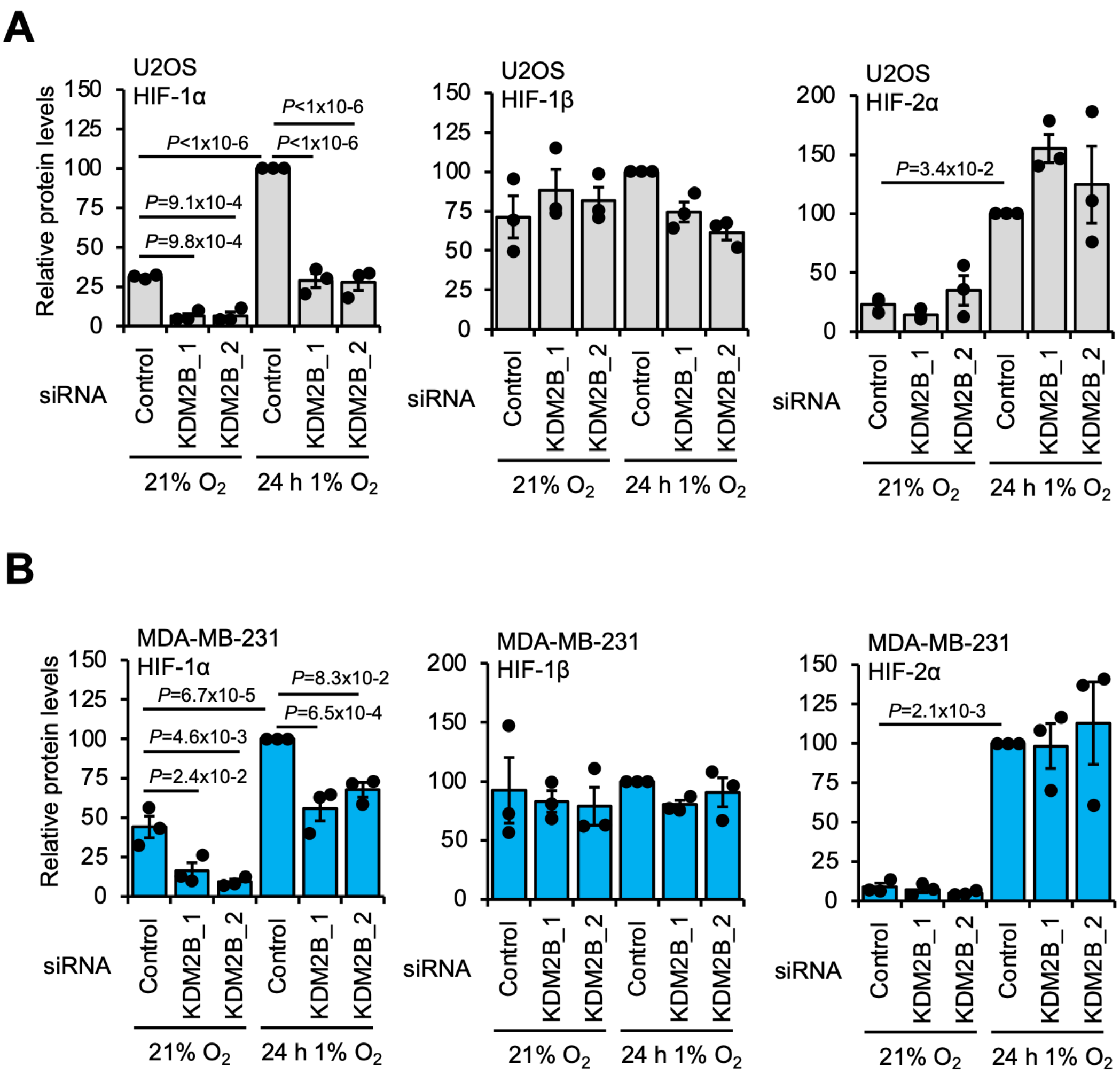

Supplementary Figure S3. Regulation of HIF levels by KDM2B in U2OS and MDA-MB-231 cells, Western blot quantification. Western blot quantification analysis for the indicated proteins in **A)** U2OS and **B)** MDA-MB-231 cells cultured at 21% oxygen, transfected with control, KDM2B_1, or KDM2B_2 siRNA for 48 hours, and exposed or not to 1% oxygen for 24 hours. β-actin was used as a normalising protein. Data represent mean (n=3 biological replicates) ± SEM. *P* values for group comparisons were calculated by one-way ANOVA with Tukey's test.

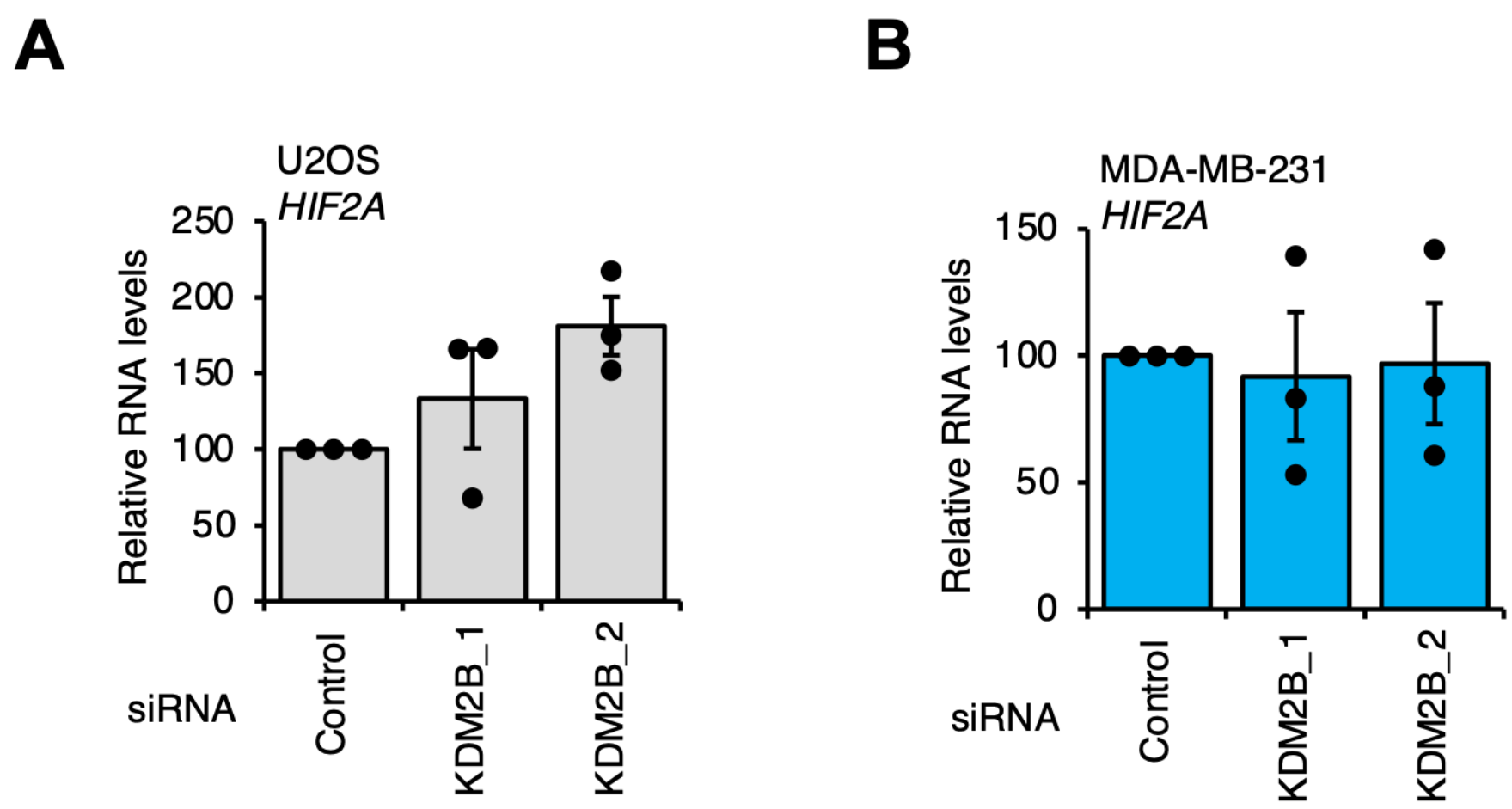

Supplementary Figure S4. *HIF2A* RNA levels in response to KDM2B depletion. qPCR analysis of *HIF2A* RNA levels in **A)** U2OS and **B)** MDA-MB-231 cells cultured at 21% oxygen and transfected with control, KDM2B_1, or KDM2B_2 siRNA for 48 hours. *ACTB* was used as a normalising gene. Data represent mean (*n=3* biological replicates) ± SEM. *P* values for group differences were calculated by one-way ANOVA with Dunnett's test.

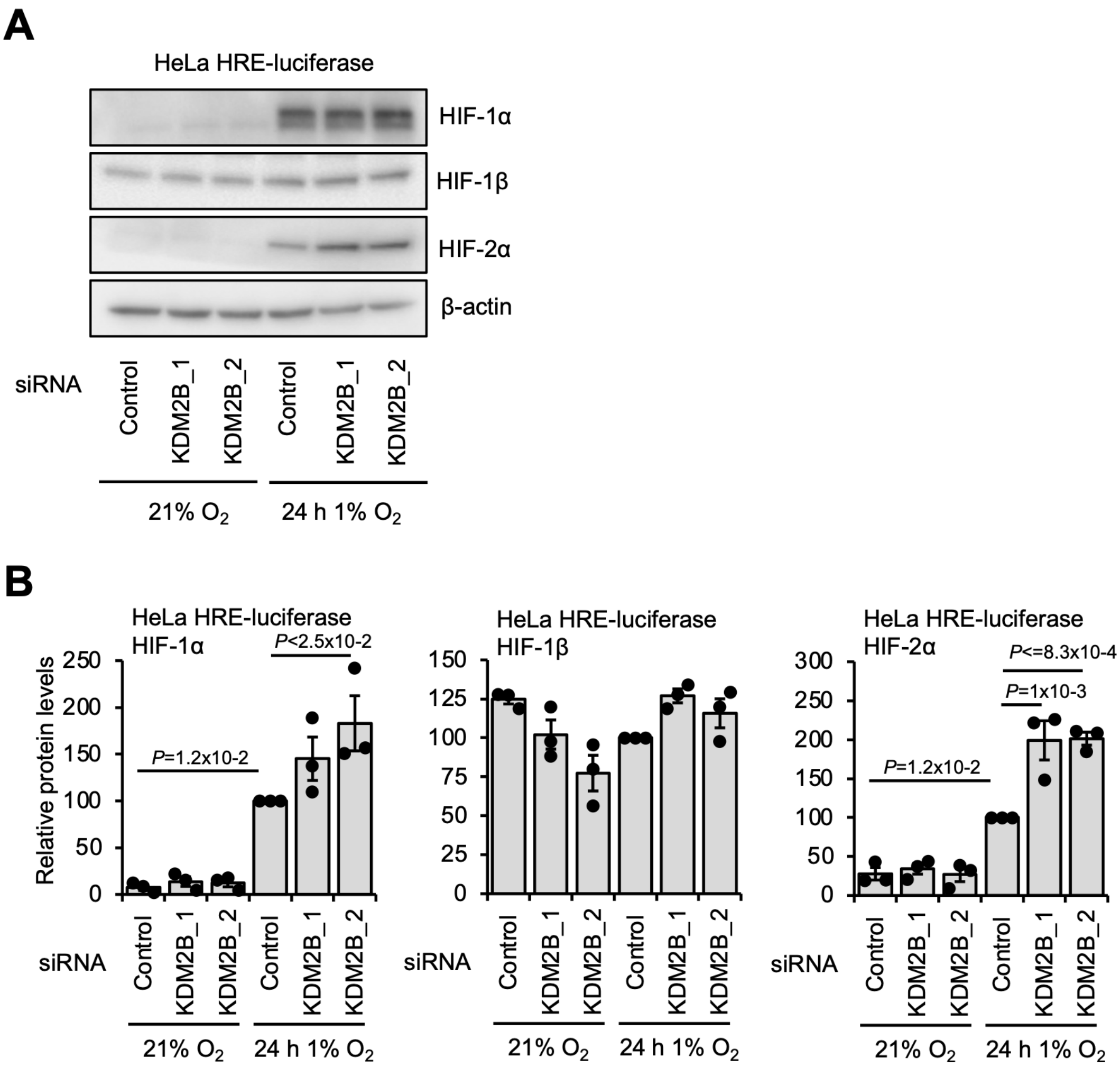

Supplementary Figure S5. Regulation of HIF levels by KDM2B in HeLa HRE-luciferase cells. **A)** Western blot and **B)** Western blot quantification analysis for the indicated proteins in HeLa HRE-luciferase cells cultured at 21% oxygen, transfected with control, KDM2B_1, or KDM2B_2 siRNA for 48 hours, and exposed or not to 1% oxygen for 24 hours. β-actin was used as a normalising protein. A representative Western blot of three biological replicates is shown. Data represent mean (n=3 biological replicates) ± SEM. *P* values for group comparisons were calculated by one-way ANOVA with Tukey's test.

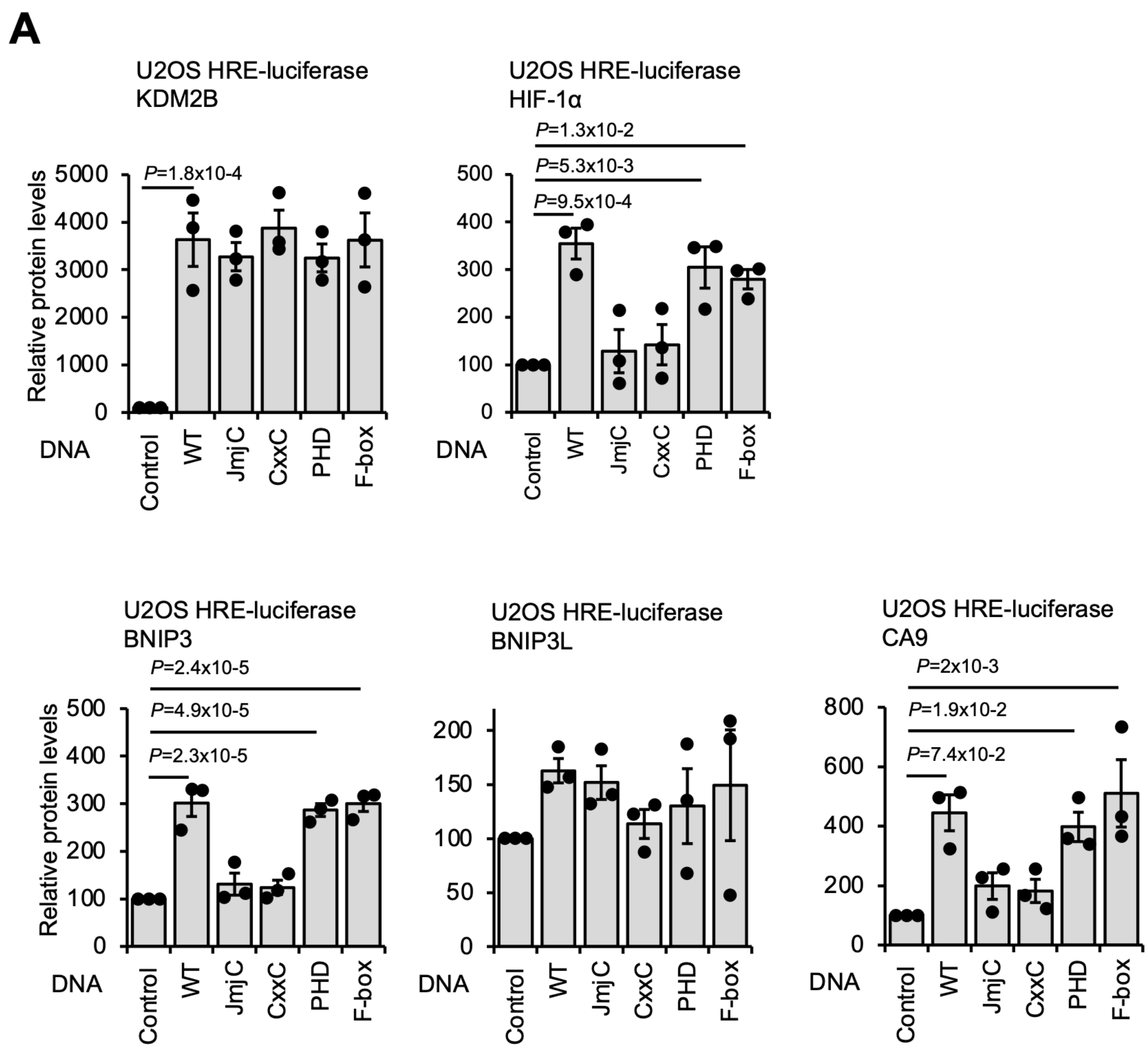

Supplementary Figure S6. KDM2B controls HIF activity through its JmjC and CxxC domains, U2OS HRE-luciferase Western blot quantification. A) Western blot quantification analysis for the indicated proteins in U2OS HRE-luciferase cells cultured at 21% oxygen and transfected with control, KDM2B WT, KDM2B JmjC, KDM2B CxxC, KDM2B PHD, or KDM2B F-box plasmids for 48 hours. β-actin was used as a normalising protein. Data represent mean (n=3 biological replicates) ± SEM. *P* values for group comparisons were calculated by one-way ANOVA with Dunnett's test.

A

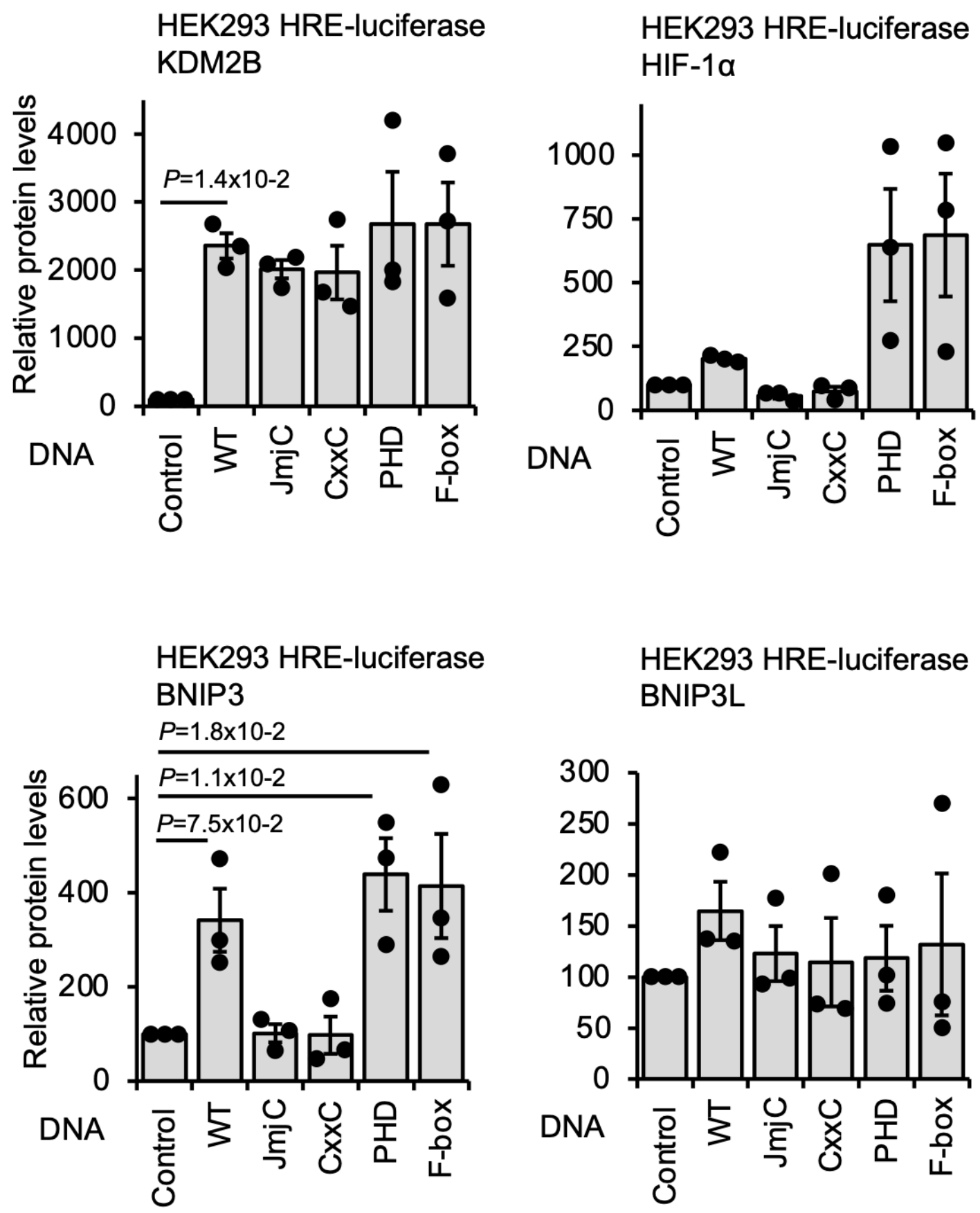

Supplementary Figure S7. KDM2B controls HIF activity through its JmjC and CxxC domains, HEK293 HRE-luciferase Western blot quantification. A) Western blot quantification analysis for the indicated proteins in HEK293 HRE-luciferase cells cultured at 21% oxygen and transfected with control, KDM2B WT, KDM2B JmjC, KDM2B CxxC, KDM2B PHD, or KDM2B F-box plasmids for 48 hours. β-actin was used as a normalising protein. Data represent mean (n=3 biological replicates) ± SEM. *P* values for group comparisons were calculated by one-way ANOVA with Dunnett’s test.

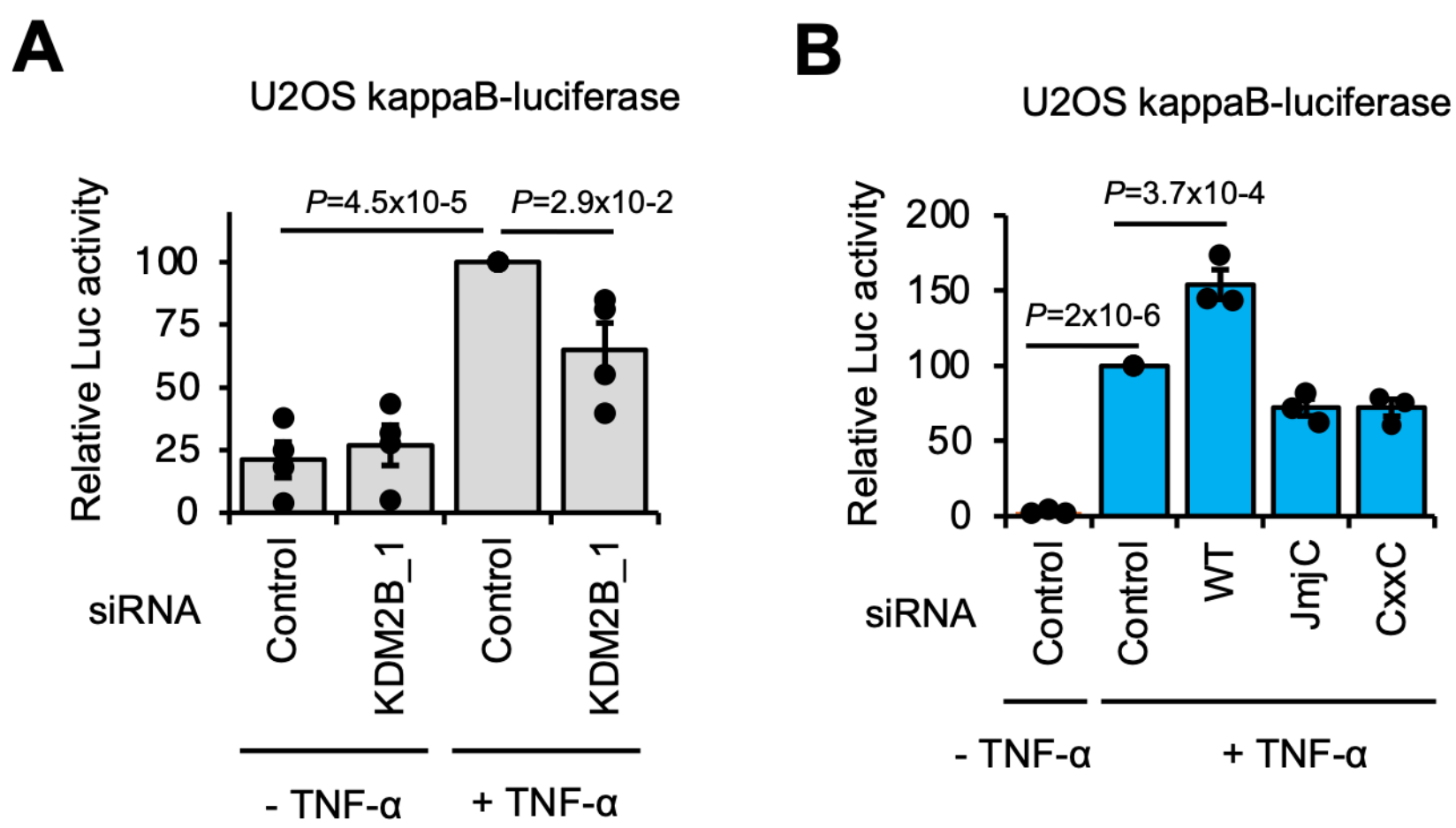

Supplementary Figure S8. KDM2B regulates NF-κB reporter activity through its JmjC and CxxC domains. A) Luciferase assay analysis in U2OS kappaB-luciferase cells cultured at 21% oxygen, transfected with control or KDM2B_1 siRNA for 48 hours, and treated with or without 10 ng/µL TNF-α for 8 hours. Data represent mean (n=4 biological replicates) ± SEM. *P* values for group differences were calculated by one-way ANOVA with Tukey's test. **B)** Luciferase assay analysis in U2OS kappaB-luciferase cells cultured at 21% oxygen, transfected with control, KDM2B WT, KDM2B JmjC, or KDM2B CxxC plasmids for 48 hours, and treated with or without 10 ng/µL TNF-α for 8 hours. Data represent mean (n=3 biological replicates) ± SEM. *P* values for group comparisons were calculated by one-way ANOVA with Tukey's test.

A

Cancer Type	*R* value	*P* value
ACC	0.34	2.2x10-3
BRCA	0.2	3.1x10-11
CESC	0.15	7.9x10-3
COAD	0.2	1.1x10-3
DLBC	0.6	1x10-5
ESCA	0.22	2.3x10-3
HNSC	0.13	2.1x10-3
KICH	0.47	6.5x10-5
KIRC	0.34	2.2x10-15
KIRP	0.28	1.9x10-6
LAML	0.17	2.5x10-2
LGG	0.09	4.1x10-2
LIHC	0.43	0
LUAD	0.11	1.2x10-2
OV	0.49	0
PAAD	0.39	9.8x10-8
PCPG	0.24	1.3x10-3
PRAD	0.33	1.5x10-2
READ	0.44	1.1x10-5
SKCM	0.25	9.3x10-8
UCEC	0.25	8.3x10-4
UCS	0.28	3.8x10-2
UVM	0.75	3.6x10-15

B

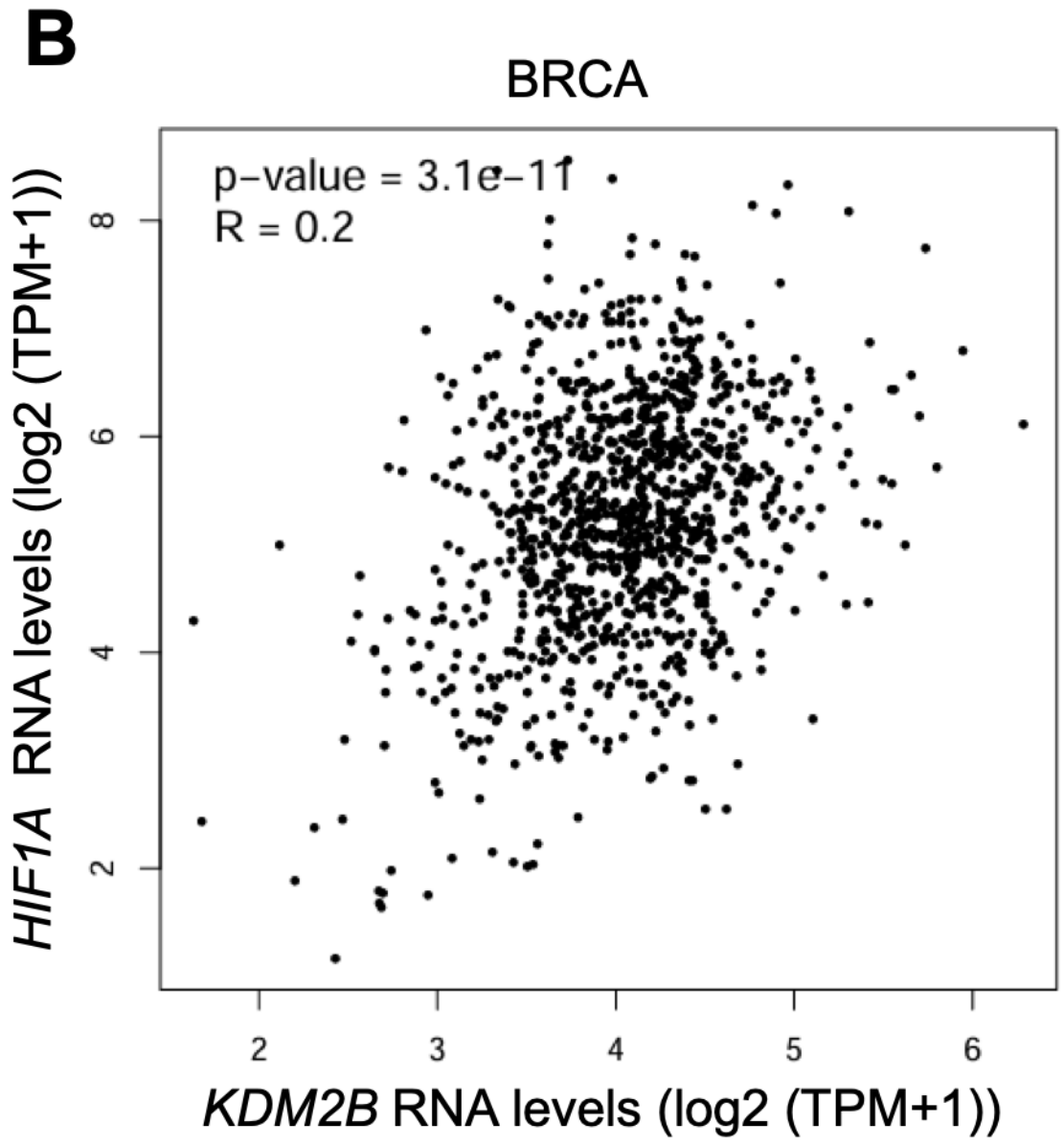

C

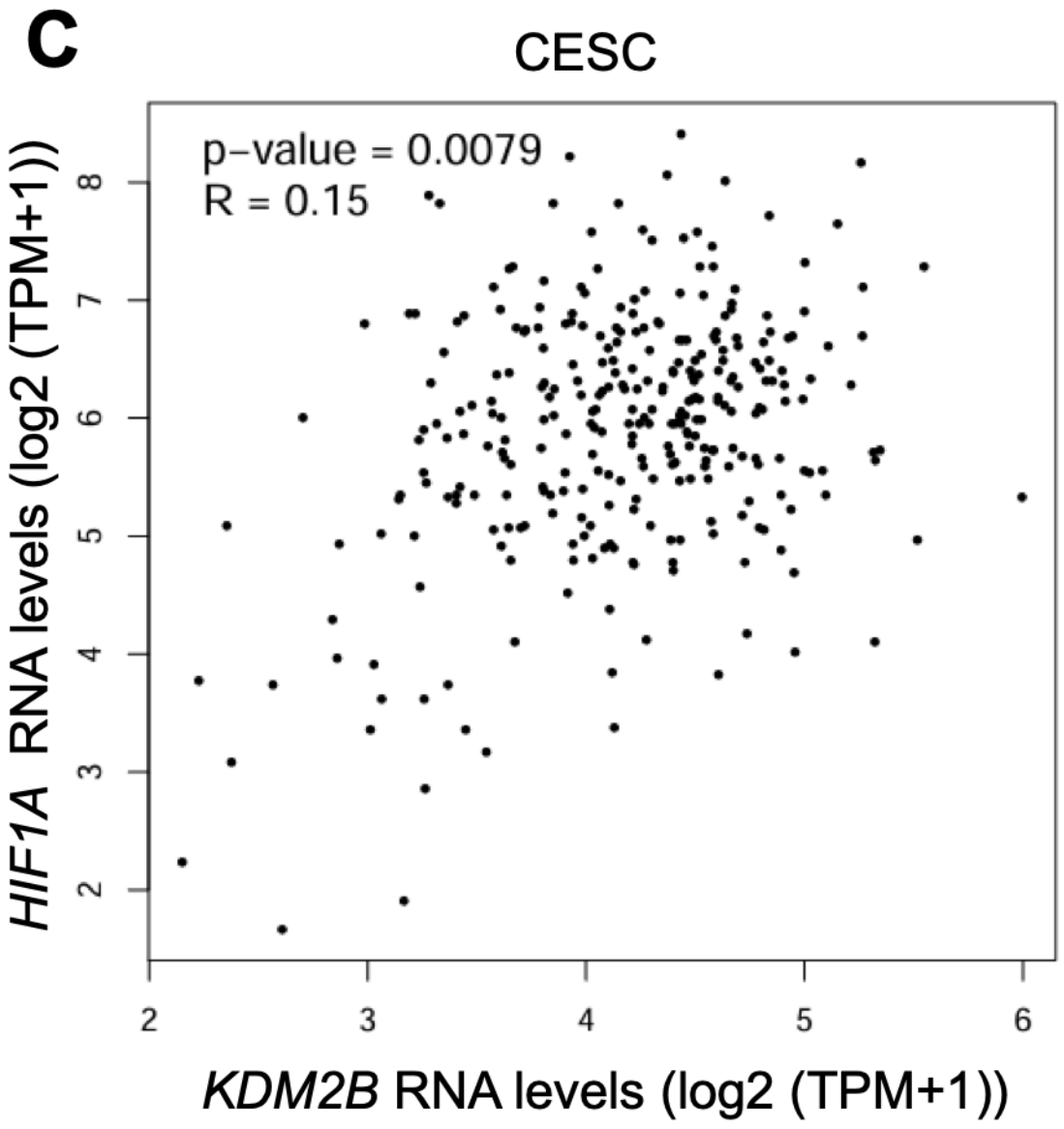

Supplementary Figure S9. Correlation analysis between *KDM2B* and *HIF1A* RNA expression in patient cancer samples. A) Correlation analysis between *KDM2B* and *HIF1A* RNA expression in patient samples from different cancer types from The Cancer Genome Atlas (TCGA) using GEPIA2, cancer types with a *P* value below 0.05 are shown. *P* values and *R* values were calculated using Pearson's correlation coefficient. Correlation analysis plots for **B)** BRCA (Breast Invasive Carcinoma) and **C)** CESC (Cervical Squamous Cell Carcinoma and Endocervical Adenocarcinoma). Cancer types=ACC-Adrenocortical Carcinoma; BRCA-Breast Invasive Carcinoma; CESC-Cervical Squamous Cell Carcinoma and Endocervical Adenocarcinoma; COAD-Colon Adenocarcinoma; DLBC-Lymphoid Neoplasm Diffuse Large B-cell Lymphoma; ESCA-Esophageal Carcinoma; HNSC-Head and Neck Squamous Cell Carcinoma; KICH-Kidney Chromophobe; KIRC-Kidney Renal Clear Cell carcinoma; KIRP-Kidney Renal Papillary Cell Carcinoma; LAML-Acute Myeloid Leukemia; LGG-Brain Lower Grade Glioma; LIHC-Liver Hepatocellular Carcinoma; LUAD-Lung Adenocarcinoma; OV-Ovarian Serous Cystadenocarcinoma; PAAD-Pancreatic Adenocarcinoma; PCPG-Pheochromocytoma and paraganglioma; PRAD-Prostate Adenocarcinoma; READ-Rectum Adenocarcinoma; SKCM-Skin Cutaneous Melanoma; UCEC-Uterine Corpus Endometrial Carcinoma; UCS-Uterine Carcinosarcoma; UVM-Uveal Melanoma.

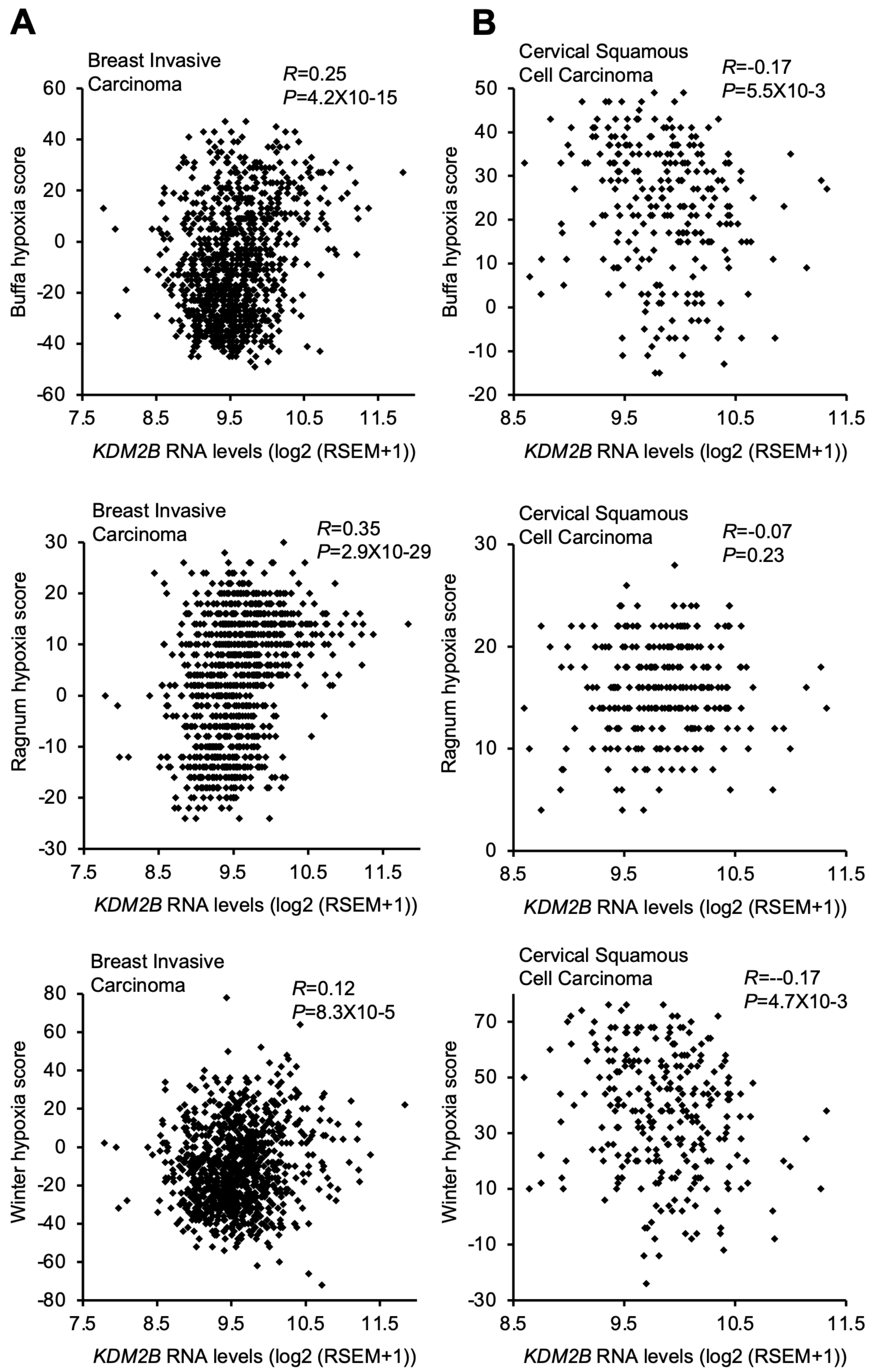

Supplementary Figure S10. Correlation analysis between *KDM2B* and hypoxia signature RNA expression in Breast Invasive Carcinoma and Cervical Squamous Cell Carcinoma. Correlation analysis plots between *KDM2B* and hypoxia signature (Buffa, Ragnum, and Winter) RNA expression in **A)** Breast Invasive Carcinoma and **B)** Cervical Squamous Cell Carcinoma patient samples from The Cancer Genome Atlas (TCGA) PanCancer Atlas using cBioPortal. *P* values and *R* values were calculated using Pearson's correlation coefficient.
